# Barriers and Facilitators to a Task-Shifted Stroke Prevention Program for Children with Sickle Cell Anemia in a Community Hospital: A Qualitative Study

**DOI:** 10.21203/rs.3.rs-2985921/v1

**Published:** 2023-07-03

**Authors:** Halima Bello-Manga, Lawal Haliru, Kudirat Ahmed, Samuel Ige, Hayatu Musa, Zainab Kwaru Muhammad-Idris, Binshak Monday, Abdulrashid M. Sani, Kemberlee Bonnet, David G. Schlundt, Taniya Varughese, Abdulkadir M. Tabari, Michael R. DeBaun, Ana A. Baumann, Allison A. King

**Affiliations:** Kaduna State University; Kaduna State University; Kaduna State University; Yusuf Dantsoho Memorial Hospital, Ministry of Health, Kaduna; Ahmadu Bello University; Kaduna State University; Ahmadu Bello University; Barau Dikko Teaching Hospital, Kaduna; Vanderbilt University; Vanderbilt University; Washington University in St Louis; Barau Dikko Teaching Hospital,Kaduna; Vanderbilt University School of Medicine; Washington University in St Louis; Washington University in St Louis

**Keywords:** sickle cell anemia, stroke, transcranial Doppler, task shifting, implementation

## Abstract

**Background:**

Children with sickle cell anemia (SCA) are at high risk for stroke. Protocols for stroke prevention including blood transfusions, screening for abnormal non-imaging transcranial Doppler (TCD) measurements, and hydroxyurea therapy are difficult to implement in low-resource environments like Nigeria. This study aimed to examine the contextual factors around TCD screening in a community hospital in Nigeria using qualitative interviews and focus groups.

**Methods:**

We conducted focus groups with health care providers and interviews with administrative leadership of the community hospital. Interview guides and analysis were informed by the Consolidated Framework for Implementation Research (CFIR) framework. Transcripts were coded and analyzed using an iterative deductive (CFIR)/Inductive (transcribed quotes) qualitative methodology.

**Results:**

We conducted two focus groups and five interviews with health care workers (nurses and doctors) and hospital administrators, respectively. Themes identified key elements of the inner setting (clinic characteristics, resource availability, implementation climate, and tension for change), characteristics of individuals (normative, control, and behavioral beliefs), and the implementation process (engage, implement, and adopt), as well as factors that were influenced by external context, caregiver needs, team function, and intervention characteristics. Task shifting, which is already being used, was viewed by providers and administrators as a necessary strategy to implement TCD screening in a clinic environment that is overstressed and under-resourced, a community stressed by poverty, and a nation with an underperforming health system.

**Conclusion:**

Task shifting provides a viable option to improve health care by making more efficient use of already available human resources while rapidly expanding the human resource pool and building capacity that is more sustainable.

**Trial registration:**

NCT05434000

## Introduction

Stroke is a frequent complication of sickle cell anemia (SCA) that is associated with increased morbidity and mortality.[1] Approximately 11% of unscreened and untreated children with SCA will have at least one stroke by 17 years of age.[2] Evidence-based practices for primary stroke prevention include screening for abnormal non-imaging transcranial Doppler (TCD) measurements coupled with regular blood transfusion therapy for at least one year, then switching to hydroxyurea therapy for an indefinite period.[3] In high-resource settings, such evidence-based practices have dropped stroke incidence rates by 92% among children with SCA.[3–5] Due to safety and availability, regular blood transfusion is not a viable option for primary stroke prevention in most resource-constrained settings, including Nigeria, where over 50% of the global 300,000 children born annually with SCA reside.[6, 7] Based on the results of our team’s randomized controlled trials (NCT01801423; NCT02560953), [8] that demonstrated efficacy in primary stroke prevention among children with SCA, the American Society of Hematology (ASH) Central Nervous System Guidelines recommend hydroxyurea for children with SCA and abnormal TCD measurements who live in resource-constrained settings where regular blood transfusions are not readily available.[9]

Before the stroke prevention trials in Nigeria, no TCD screening was performed in Kaduna State, Nigeria, where an estimated 20,040 children with SCA reside. After completing enrolment for the SPRING trial, as standard care, we initiated TCD screening for children with SCA in our center, but only 10.2% (2,057) of the estimated eligible children with SCA in Kaduna have been screened. To increase the reach of TCD screening, we obtained funding from the Fogarty International Center at the NIH to replicate our stroke prevention program, which was based in an academic center, in a community hospital in a densely populated part of Kaduna, where most patients with sickle cell disease (SCD) seek care. [10] After completion of the SPRING trial, to expand the reach of TCD screening for children in a resource-constrained area, nurses were trained by radiologists to complete the TCD screening. This task shifting [11]where providers with different roles are trained to perform a single health-related task, from radiologists to nurses was successful, as the nurses in the academic setting assumed the role of TCD assessments.[10] The stroke prevention program includes TCD screening and treatment with hydroxyurea, a daily oral medication, if the TCD measure is elevated. Education of medical staff and families along with a better understanding of the community hospital and its external environment are crucial to providing these services.

To better understand how to replicate the stroke prevention program in a community hospital as opposed to an academic setting, we conducted a qualitative study using the Consolidated Framework for Implementation Research (CFIR) as a guiding framework.[12] CFIR is an implementation science determinant framework that can be used to help understand the contextual factors of TCD screening through a menu of constructs of five domains: (1) intervention characteristics, (2) inner setting (3) outer setting, (4) characteristics of individuals, and (5) implementation process. We used a qualitative approach to understand the nuances of implementing TCD screening in a community hospital setting to inform future efforts to increase the reach of TCD screening and stroke prevention in resource-limited settings where the burden of SCA and stroke is high.

## Methods

### Study Design and Site

This prospective qualitative study received institutional review board approval from the Kaduna State Ministry of Health Ethics Committee (MOH/ADM/744/VOL.1/920). The study was conducted at Yusuf Dantsoho Memorial Hospital, a 100-bed-capacity community hospital with an annual hospital attendance of 144,000. The research team met with hospital leadership prior to initiating study recruitment to introduce the importance of stroke prevention strategies including task shifting. Thereafter, the matron of the pediatric unit was identified as the liaison between the study team and the hospital.

### Recruitment

We conducted focus group discussions with health care providers (i.e., doctors and nurses) and key informant interviews with administrative staff of the hospital. Written informed consent was obtained from participants. Focus groups and Interviews were led by a PhD-level Researcher and 2 note takers. Focus group participants were identified and selected from the pediatric unit of the hospital. Nurses were eligible to participate if they: (1) had worked in the pediatric unit for more than three years, and (2) were willing to participate. Doctors were eligible if they: (1) had prior experience managing children with SCD, (2) had worked in the community hospital for more than one year, and (3) were willing to participate. Key informant interviews were conducted with members of the hospital’s administrative staff who were involved in significant decisions affecting staff rotations, redeployment, and other hospital policies. They included the medical director, hospital secretary, head of pediatrics, head of radiology, chief matron, deputy matron, and the hospital accountant. Inclusion was based on their positions and willingness to participate in the study.

### Interview and Focus Group Guides

The study team developed the interview and focus group guides in English using the CFIR as a guide.[12] Topics covered by both guides, with major CFIR constructs in parentheses, included:

1. Familiarity with stroke detection and prevention in children with SCD (intervention characteristics)
2. Knowledge of TCD and its role in stroke screening (characteristics of individuals)
3. Understanding and perception of task shifting (intervention characteristics)
4. Challenges and advantages of using TCD with task shifting to develop and implement a stroke prevention program for children with SCA (implementation process)
5. Education needed for providers and for families/caregivers of children with SCA (implementation process)
6. Information and resources needed to implement and sustain a stroke prevention program in the community clinic setting (inner and outer setting)

### Procedures

The study coordinator contacted eligible health care providers and hospital administrative staff to schedule focus groups and interviews based on participant availability. Each focus group and interview began with introductions, an explanation of the purpose of the study, and a reminder that the session would be recorded. To set the stage for the group discussion and interviews, a short video on stroke in SCD was shown. Sessions lasted about 60 minutes, ending when saturation was reached. Participants were served lunch. Interviews with hospital administrative staff lasted about 45 minutes. All participants completed a short demographic survey before the focus group or interview detailing their work experience.

### Audio Recording and Transcription

Focus groups and administrator interviews were audio recorded using a digital recording device. Audio les were shared with the Vanderbilt University Qualitative Research Core (VU-QRC) for data analysis using secure transfer technology. The audio les were professionally transcribed using a fast online service.[13]

### Development of a Coding System

The VU-QRC managed qualitative data and analysis, led by a PhD-level psychologist. Data coding and analysis were conducted by following the COREQ guidelines.[14] A hierarchical coding system was developed and refined using the CFIR framework, the focus group/interview guides, and a preliminary review of transcripts. The top-level categories of the coding system, with major CFIR construct in parentheses, were:

1. clinical/patient factors (characteristics of individuals),
2. life impacts (characteristics of individuals),
3. hospital/organization setting (inner setting and outer setting),
4. individual characteristics (characteristics of individuals),
5. intervention characteristics (intervention characteristics),
6. implementation process (process of implementation),
7. education and outreach (process of implementation),
8. barriers and facilitators (all CFIR constructs),
9. clarification (all CFIR constructs),
10. contingency (all CFIR constructs),
11. example experiences (all CFIR constructs),
12. world events (outer setting), and
13. provider/health team member (characteristics of individuals).

Major categories were further divided from one to 12 subcategories, with some subcategories having additional levels of hierarchical division. Definitions and rules were written for the use of coding categories. The coding system underwent seven iterations before the final version was accepted by the team.

### Coding the Transcripts

Experienced qualitative coders first established reliability by using the coding system on two transcripts and resolving any discrepancies. They then independently coded the remaining transcripts. Each statement was treated as a separate quote and could be assigned up to 14 different codes. Transcripts were combined and sorted by code. Transcripts, quotations, and codes were managed using Microsoft Excel 2016 and SPSS version 27.0. Analysis was conducted using an analytic spreadsheet with all of the applied codes, the associated quotes, and any contextual text (e.g., moderator’s question) needed to understand the quote.

### Processing Coded Transcripts

We used an iterative inductive/deductive approach to qualitative analysis,[15–18] resulting in a conceptual framework. Inductively, we sorted the coded quotes by coding category to identify higher-order themes and relationships between themes. Deductively, we were guided by the study questions, the CFIR, [12, 19, 20] and the theory of planned behavior.[21, 22] The theory of planned behavior was added to the coding analysis to provide a more detailed understanding of the motivations, beliefs, and behaviors of the individuals involved in program implementation. The process was iterative in that the conceptual framework was theoretically informed, while the specific framework content was derived from the coded qualitative data. The final iterations were reviewed by an implementation scientist and the team until all research team members reached agreement.

## Results

### Participants

Two focus groups were conducted at the community hospital with seven participants in one and 12 participants in the other. The mean age of health care providers was 39 (SD 10.0). Three providers were male, and 16 were female. The average years of work experience was 12.8 (SD 9.9). The first focus group had six doctors and one nurse (labeled “doctors focus group”). The second group consisted of all nurses and nurse midwives (labeled “nurses focus group”). Four tribes were represented: Igala, Yoruba, Hausa, and Edo. Five administrators were interviewed, with a mean age of 52.8 (SD 3.3). Two administrators were male, and three were female. The average years of work experience was 21.4 (SD 7.8). Administrators included two nurses, two doctors, and one accountant. Three tribes were represented: Hausa, Yoruba, and Bajju.

### Conceptual Framework

**Figure 1:**
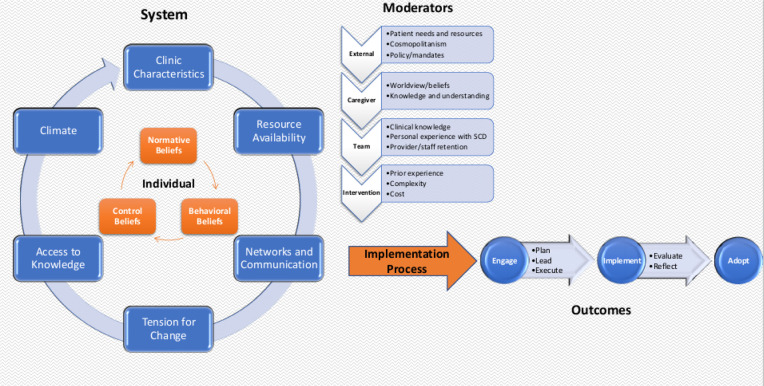
Factors influencing the adaption of TCD task shifting

Figure 1 presents the conceptual framework we created following our qualitative analysis. The outer circle, largely inspired by the CFIR framework inner setting constructs,[12] represents elements of the health care setting and sources of influence that drive the adoption of a stroke prevention strategy implemented using TCD screening and task shifting. The inner circle, influenced by the outer circle and inspired by the theory of planned behavior,[21] represents the individual attitudes, beliefs, and behaviors of the health care providers involved in planning and implementing the stroke prevention program. The nested circles and arrows represent the dynamic interaction of systems and individual-level characteristics. The central area of the diagram represents modifying factors that can influence the implementation process. These include external factors (outer setting), caregiver characteristics (individual characteristics), implementation team characteristics (individual characteristics), and the task shifting intervention (intervention design). The joint operation of providers within a health system leads to the implementation process. All of the activities are related to implementing the plan, which is depicted as having three stages: (1) engaging, (2) implementing, and (3) adopting the prevention program.

In the following sections, each element of the framework is described and supported using quotes from coded transcripts, which are organized in Table 1. Each quote is labeled by the source of the quote (i.e., doctors focus group, nurses focus group, or administrator).

### System

For the health care system (inner setting), we identified six interacting themes: (1) clinic characteristics, (2) resource availability, (3) within-setting communication, (4) tension for change, (5) accessibility of information, and (6) implementation climate.

#### Clinic characteristics.

Participants discussed how local clinics are organized to provide care and what kind of care is provided. One of the important features of clinics is how the different disciplines, such as medicine and nursing, are organized and represented. Nurses tended to have rotations, while physicians tended to have primary assignments. One nurse described how task shifting was already occurring in the clinic because of a shortage of doctors:

“Now that we have shortage of the medical doctors. Most times, if you come to this hospital on Tuesdays and Thursdays when they see the diabetic and hypertension patients, the nurses are also involved…in seeing the patients, because the doctors that are here cannot cover all the patients…so that nobody will be left behind.” (Nurses’ Focus Group)

#### Resource availability.

Many participants discussed a lack of resources. Resource gaps may be due to lack of money, low pay, failure to pay temporary staff, insufficient staffing, and staff turnover. Nurses talked about how high turnover among nurses is created by low pay, and how turnover increases caseloads and generates stress as (Table 1).

#### Networks and Communications.

This refers to the formal and informal communications within an organization. The need to involve all stakeholders from the beginning of the program was stressed by one of the administrators:

“The Medical Director [MD] has his management team, which all the head of departments are part of it. They have management meetings actually every month or twice. So, everybody has to [inaudible] be told exactly this is what is happening and expect…That’s why I said the Ministry should be aware, then the MD, then his management, then it drops to staffs and then community.” (Administrator)

#### Tension for change.

Tension for change is the degree to which staff see the current conditions as problematic and in need of change. The greatest tension for change was around staffing problems. According to one administrator, task shifting was necessary and already being implemented in other areas of hospital functioning. Thus, the administrator felt that the hospital would be ready to add the proposed stroke prevention program (Table 1). This nurse was also ready to embrace task shifting:

“Without the task shifting, the workload will be too much on one person, like what we are experiencing here in our facility. We only have one radiologist, but with this task shifting, when others are being trained, you will see that they’ll be able to attend to more number of children, than delaying, waiting for only one radiologist to take care of the investigation or the test.” (Nurses focus group)

#### Accessibility of information.

The importance of access to knowledge about SCA and stroke for both the health care providers and parents was highlighted as an important factor in the success of the program and improving the care of children with SCA. To be useful, appropriate information about the intervention and the role changes involved in task shifting, along with accurate information for the family’s needs, should be readily available. This nurse stressed the importance of readily available training for nurses on appropriate diagnosis:

“Another challenge is also the accuracy of diagnosing these cases, because of inadequate knowledge or training the nurses acquire (Nurses focus group).

Similarly, an administrator talked about the importance of all team members having access to training on how to use the Doppler machine:

“So, all you need to do is to train what to do with the machine. So, in this place if somebody [inaudible] you could go downstairs, I’m sure if you use it once or three times is easy to pick. Yeah. But we have a GE machine which is power controlled. (Administrator)

#### Implementation climate.

Implementation climate is the willingness of staff to change, the degree to which change is part of the local culture, the recognition of the importance of change, and the extent to which participation in change efforts is encouraged and rewarded. The participants showed some evidence of an organizational climate to support change. One nurse discussed the importance of planning for change:

“Like somebody talked about planning, somebody said something about planning. Even if it’s [your] own housework, if you don’t plan how to do it…at the end of the day, you will leave some things undone. Some simple, simple things that you’re supposed to do, [inaudible] you’ll leave them undone.” (Nurses focus group)

#### Summary of system.

The discussion that focused on the clinic and health care system suggested that the local system is burdened by high patient load, lack of funding, low pay, inadequate staffing, staff rotation, and high turnover. Despite these problems, there is evidence that, out of necessity, staff are willing to engage or are already engaging in task shifting and that there is a commitment among staff to identify permanent changes, including access to training and improved communication, to better meet the needs of patients, providers, and the clinic.

### Individual

The inner circle labeled “individual,” while part of the CFIR, is enhanced by using the theory of planned behavior.[21] The three elements of the theory are normative beliefs, control beliefs, and behavioral beliefs. Normative beliefs are the individual provider’s understanding of the behavioral norms (how people are expected to behave) that are part of their institution or profession. Behavioral beliefs are ideas about what to do and how likely different behaviors are to lead to desired outcomes. Control beliefs refer to an individual’s sense of the ease or difficulty of performing different behaviors.

#### Normative beliefs.

The discussion of what is usually done focused largely on task shifting the work from doctors to nurses. Doctors, nurses, and administrators all agreed that the program was important and that task shifting was already the norm (See Table 1).

“Denitely, this hospital normally we practice task shifting. Because in Nigeria, even if you look at the....Not even radiologist, even the ratio of the doctors to the patients—already there’s task shifting in the nurses. And even in radiology, there’s task shifting with radiographers and sonographers, because the amount of patients you see as a radiologist, it’s not possible for you to see.” (Administrator)

#### Behavioral beliefs.

Behavioral beliefs were focused on the screening procedures and on having an idea of what would be successful. One nurse talked about how to engage caregivers of children with SCA to encourage success (see Table 1). Behavioral beliefs also involve understanding what the outcomes or benefits of a choice are. This doctor described the benefits of adopting the screening program:

“Of course, it will reduce the workload of the doctors. That’s the one important benefit I see for the hospital. It will reduce the workload…I think is a plus for the hospital.” (Doctors focus group)

According to administrators, task shifting is the only possible solution. They also saw the program as a benefit for the mothers and caregivers of children with SCA, because it would give them more insight into the disease. One administrator was certain that the stroke prevention program would have positive outcomes for all involved:

“In fact, this is a very good program. It will go a very long way because the patients will benefit from it. The person that is carrying out the work will also benefit from it, because it is a knowledge being given to him.… Also, the hospital will benefit from it because many people would like to come in order to get a better service.” (Administrator)

#### Control beliefs.

Control beliefs are how confident one is in performing a specific behavior. In the focus group of doctors, the focus of discussion on control beliefs was on the nurses’ ability to learn and perform the task shifting activities. According to this administrator, the stroke prevention program will work:

“It will work if we put hands together.” (Administrator)

This nurse was confident that nurses would be able to handle task shifting:

“The patient will always be attended to. Because like now, we are always available. The nurses are always on ground” (Nurses focus group)

#### Summary of individual.

For the stroke prevention program to be adopted, the individual clinicians involved need to believe that it is the right thing to do, they need to know what to do, and they need to be confident that they can implement the program. There is evidence that at least some of the doctors, administrators, and nurses are ready to adopt the program.

### Implementation Process

Once an organization has decided to implement a stroke prevention program, according to CFIR, there is an implementation process that is followed. We conceptualized the process as having three stages. First, an implementation team is **engaged** and works to develop goals, policies, personnel, time schedules, and other details of what the program will look like. This involves planning, leading, and executing.[23]The next step is to **implement** the program. Once a program has been implemented, it may eventually transition to the final stage, which is **adoption**. In the adoption stage, the focus is on sustainability and creating positive outcomes for the prevention strategy.

#### Engage.

To implement the proposed stroke prevention program, several different parties in both the inner and outer setting must be engaged, including the Ministry of Health, doctors, nurses, caregivers, and the community at large. Some of the quotes include:

“The need, especially the pediatricians, I think they should be the frontline.” (Doctors focus group)

“Giving health talks. In church and any other religious. You can also go through that route because the people tend to listen to them.” (Doctors focus group)

There was also discussion of how the Ministry of Health and Commissioner need to be involved:

“This is a policy now; policies are done from the ministry. You have a Commissioner, you have a Permanent Secretary. So, the hierarchy says, ‘If you want to get something, you have to apply through the Ministry of Health.’ So, the Ministry of Health are in charge of policy. And then they’ll tell you, you could go ahead and implement it. So, I believe the right thing to do is to write to the Commissioner through the Ministry, then the Ministry will tell you, ‘Fine, the thing is applicable.’” (Administrator)

#### Implement.

The implementation process consists of many parts such as hiring new staff, training existing staff, and reorganizing the way care is provided. One nurse talked about the importance of setting a program start date.

“A date should be fixed for TCD screen. Like the sickle cell clinic is every Friday. So why not that Friday that they’re coming for the clinic” (Nurses focus group)

#### Adopt.

There was limited discussion in the focus groups and interviews about adoption of the program, but comments from administrators showed that they were ready to adopt the program.

“I believe our management will support this program; even the nursing department will support this program. And since it is coming down to us, we’ll also support the program.” (Administrator)

#### Summary of implementation process.

Participants identified several important elements of the process of implementing the task shifting stroke prevention program. It is important to engage all essential stakeholders, including patient families, the government, and other external organizations. Participants identified specific ways to reach out to the community to ensure that potentially eligible families are aware of the need for stroke prevention and to advertise the program. Creating an implementation team is an important part of program planning. Selecting and training the right people is a vital aspect of team development. The implementation process should proceed from engagement to resource development to setting a start date for the program to begin.

### Moderators

Moderators are factors that influence how the program is planned, implemented, and adopted. Moderators can be either barriers, factors that make the work more difficult or complex, or facilitators, factors that contribute to the success of the work. Four main categories of moderators were identified: external (outer setting), caregiver (outer setting), teams (inner setting), and intervention (intervention process).

#### External.

Outer setting factors are things outside of the clinical setting that can have an impact on the planning and implementation of the program. Three main themes were identified: (1) patient needs and resources; (2) cosmopolitanism, which refers to external organizations; and (3) policies and mandates. Discussion of the needs and resources of patients largely focused on their financial needs, which is especially salient in Nigeria, which has a high rate of extreme [24]. One doctor identified having money for transportation as a family need.

“Finance. You can give them money; most of them will come for certain when you give them money for transport” (Doctors focus group)

This administrator talked about different types of people in the community who are considered friends of the hospital and the importance of their involvement in program planning and implementation:

“Friends of hospital, you see the Medical Director (MD), our present MD, they have a committee for friends of hospital. So, I don’t know who are directly now, but [crosstalk] traditional rulers....Yeah, traditional rulers, head of communities, and certain people who work within the hospital as volunteers as friend of hospital” (Administrator)

The relationship between primary and secondary care settings in Nigeria will influence how the program is implemented, according to this administrator:

“We’re only encouraging, because the primary health, we cannot refer the patient from the secondary health facility to the primary health facility....The only thing we normally encourage them is, let’s say the antenatal care or the immunization. Then we always encourage them that it’s not necessary for them [to] come here. If they have primary health centers in their places, let them be attending, so that at least they will reduce the workload on us. So, by the time they are attending the…if there is anything that is bigger than them, then they will refer them to come to us.” (Administrator)

This nurse thought that government policy might be used to support staffing:

“I feel that challenge can be tackled if the government too can step up to improve the manpower shortages in our hospitals.” (Nurses focus group)

#### Caregiver.

How the patients’ caregivers understand SCA, stroke risk, and stroke screening may influence utilization of the program. Parents of children with SCA are often stressed and overburdened by their child’s condition. This administrator spoke about the financial and social burden parents face:

“Parents is a burden every day, your child is sick, you’re in the hospital and he’s not doing well, and you’re wasting your money, you’re wasting your time, and the child is not improving.” (Administrator)

This nurse spoke about how to support caregivers:

“When a child is being brought with a case of sickle cell, you will see that the parents are restless. The first thing, you reassure the parents. Then the child, if he’s in pain, there are analgesics that you can give, you as a nurse.…So, you give the necessary things; you take care of the child until he is calm.” (Nurses focus group)

#### Teams.

Within the inner setting, the composition and training of the team that will plan and implement the prevention program is an important moderator. This administrator talked about the importance of all team members having common knowledge about the body:

“There are certain things, you think these are things that you did maybe from basic secondary schools, it’s emphasized, emphasized, emphasized, is not that you don’t do it, but you need to have some background of sciences, of anatomy, of physiology….How will you know all those things if you don’t comprehend?” (Administrator)

One administrator thought that the training should be focused on radiology technicians rather than nurses, because there is less turnover among radiology technicians (see Table 1). Another administrator cautioned that not everyone can be trained to task shift:

“They have to know what they are looking for, or what should they look for. If not, it’s like a pilot, everybody you’ll talk to y a plane, but you have to be regulated.…So, medicine, it’s true I agree with task shifting, but it has its own limitations.” (Administrator)

#### Intervention.

Some aspects of the stroke prevention program could create barriers to implementation. Three themes were identified: (1) prior experience, (2) complexity, and (3) cost (see Table 1). Complexity refers to the number of people, departments, and professionals who have to be trained to work together toward the goal of implementing a screening program. Cost involves the additional money or resources needed to launch and sustain stroke screening.

#### Summary of moderators.

Participants in the focus groups and interviews identified several potential barriers and facilitators that might affect the implementation of task shifting to prevent stroke in children with SCA. External barriers included patient needs and resources along with support, or lack of support, from external organizations, including the Ministry of Health, nongovernmental organizations, and friends of the hospital committees. Support can be directly provided by external organizations, or it can come indirectly from policies and mandates. Caregivers of children at risk for stroke due to SCA were seen as stressed, burdened, and in need of well-delivered education about the child’s condition and about how to navigate the health care system. Health care teams assigned to task shift must be knowledgeable, well trained, enthusiastic, and willing to work hard. Having some direct experience with SCA can be a facilitator. The intervention itself will be easier to implement if the hospital and hospital staff have prior experience with task shifting and with screening and triaging high-risk patients. Complexity and cost are potential barriers that may make it dificult to implement and sustain the stroke prevention program.

## Discussion

Given the high number of children with SCA born in Nigeria and the paucity of personnel capable of performing TCD screening for stroke prevention in community hospitals,[25]task shifting from physicians to nurses is a viable option. As a first step in improving the reach of TCD screening, we conducted a qualitative study at a community-based hospital in Kaduna State, Nigeria. We found enthusiasm for task shifting to implement a stroke prevention program. Providers and administrators (system) endorsed the importance of preventing stroke among children with SCA. Health care providers and administrators viewed the evidence-based program as an opportunity that was needed to better serve affected families. Generally, health care providers reported that they were ready and willing to adopt task shifting, and administrators stated that the hospital system was overloaded and that task shifting would relieve some of this burden.

Both the system and individual themes captured the stress that exists in providing health care in a community hospital where requested medical and nursing services are greater than the capacity to deliver these services. High patient volume and staffing shortages of both physicians and nurses strain the system. Meager salaries were identified as one of the barriers and a common reason for staff turnover. Even with rotating or visiting nurses, health care providers endorsed task shifting and acknowledged that the approach was already being used for other clinical challenges. Both doctors and nurses endorsed task shifting TCD screening to nurses to better serve patients. Several providers identified that task shifting was necessary and the only way a limited number of health care providers could adequately care for the large population of individuals with SCD. A limitation in the task shifting strategy from physicians to nurses is the inadequate staffing of both professional health care providers. Therefore, task shifting TCD screening to radiology technicians was identified as a viable and potentially more sustainable solution. In the United States, physicians and nurses rarely perform TCD screening.[26]

A common facilitator to implementing stroke prevention programming is providing education to health care providers and families. Given the high turnover rates of nurses, we anticipate frequent educational sessions and linkage to the already established secondary and tertiary health care centers. A systematic approach to training and skills checks must be established to ensure fidelity of TCD screenings. Ideally, a senior staff member would serve as the champion and would also be able to “train the trainer” for future hires. To reach families, we plan on in-person advocacy through community meetings in town halls and religious gatherings and media campaigns over the radio, on social media, and in other venues to inform caregivers about the importance of TCD screening to prevent stroke. Consistency and bundling of services (i.e., TCD screens always conducted on Fridays during usual SCD clinic) may facilitate caregiver buy-in.

Costs associated with services and the financial burden for families must also be considered for implementation and long-term adaptation of the stroke prevention program. To deliver some of this care closer to home, an extension of TCD screening may be needed in the primary care setting. This would decrease costs for families. Serial educational and booster training sessions will be needed to maintain skills of TCD screening at all of the sites, in the community hospital or primary care.

Support and buy-in from the Ministry of Health was also identified as a necessary component to change policy related to health care delivery and staffing. Within the state of Kaduna and the country of Nigeria, the Ministry of Health has great influence over mandated evidence-based practices. There is a huge deficit in human resources for health in Nigeria. Currently, the World Health Organization estimates four doctors per 10,000 people in Nigeria, compared to 26 doctors per 10,000 people in the United States.[27, 28] These deficits are replicable across other health care personnel, including nurses. Even if Nigeria embarks on an emergency training of doctors who will offer stroke prevention to children with SCA, it will take six years to certify general medical doctors and another four years to train specialists (radiologists, hematologists). Clearly, other alternatives are needed to address this shortage of health personnel. Task shifting provides a viable option to improve health care by making more efficient use of already available human resources[29] while rapidly expanding the human resource pool and building capacity that is more sustainable.[30]

## Conclusion

Task shifting provides a viable option to improve health care by making more efficient use of already available human resources while rapidly expanding the human resource pool and building capacity that is more sustainable. Health care providers and administrators at a community hospital in Kaduna, Nigeria, endorsed the importance of stroke prevention among children with SCA. Physicians and nurses were open to task shifting for completing TCD, as many providers have already adopted this practice for other needs due to short staffing. Repeated cycles of education will be needed for all levels of the medical team and the families. For these efforts to yield the expected favorable outcomes, the government of Nigeria must recognize the impact of SCA on its population and make deliberate efforts toward improving care for individuals with SCA by enabling policies that will be implemented into routine practice.

## Data Availability

Available on request.

## Abbreviations

CFIR: Consolidated Framework for Implementation Research
SCA: sickle cell anemia
SCD: sickle cell disease
TCD: transcranial Doppler
VU-QRC: Vanderbilt University Qualitative Research Core
